# Peripheral glucocorticoid receptor antagonism by relacorilant with modest HPA axis disinhibition

**DOI:** 10.1530/JOE-22-0263

**Published:** 2022-12-22

**Authors:** Eva M G Viho, Jan Kroon, Richard A Feelders, René Houtman, Elisabeth S R van den Dungen, Alberto M Pereira, Hazel J Hunt, Leo J Hofland, Onno C Meijer

**Affiliations:** 1 Department of Medicine, Division of Endocrinology, Leiden University Medical Center, Leiden, the Netherlands; 2 Einthoven Laboratory for Experimental Vascular Medicine, Leiden University Medical Center, Leiden, the Netherlands; 3 Department of Internal Medicine, Division of Endocrinology, Erasmus Medical Center, Rotterdam, the Netherlands; 4 Precision Medicine Lab, Oss, the Netherlands; 5 Department of Endocrinology and Metabolism, Amsterdam University Medical Center, Amsterdam, the Netherlands; 6 Corcept Therapeutics, Menlo Park, CA, USA

**Keywords:** glucocorticoid receptor, HPA axis, pituitary, Cushing’s syndrome

## Abstract

Glucocorticoid stress hormones are produced in response to hypothalamic–pituitary–adrenal (HPA) axis activation. Glucocorticoids are essential for physiology and exert numerous actions via binding to the glucocorticoid receptor (GR). Relacorilant is a highly selective GR antagonist currently undergoing a phase 3 clinical evaluation for the treatment of endogenous Cushing’s syndrome. It was found that increases in serum adrenocorticotropic hormone (ACTH) and cortisol concentrations after relacorilant treatment were substantially less than the increases typically observed with mifepristone, but it is unclear what underlies these differences. In this study, we set out to further preclinically characterize relacorilant in comparison to the classical but non-selective GR antagonist mifepristone. In human HEK-293 cells, relacorilant potently antagonized dexamethasone- and cortisol-induced GR signaling, and in human peripheral blood mononuclear cells, relacorilant largely prevented the anti-inflammatory effects of dexamethasone. In mice, relacorilant treatment prevented hyperinsulinemia and immunosuppression caused by increased corticosterone exposure. Relacorilant treatment reduced the expression of classical GR target genes in peripheral tissues but not in the brain. In mice, relacorilant induced a modest disinhibition of the HPA axis as compared to mifepristone. In line with this, in mouse pituitary cells, relacorilant was generally less potent than mifepristone in regulating *Pomc* mRNA and ACTH release. This contrast between relacorilant and mifepristone is possibly due to the distinct transcriptional coregulator recruitment by the GR. In conclusion, relacorilant is thus an efficacious peripheral GR antagonist in mice with only modest disinhibition of the HPA axis, and the distinct properties of relacorilant endorse the potential of selective GR antagonist treatment for endogenous Cushing’s syndrome.

## Introduction

Glucocorticoid (GC) stress hormones are essential for physiology and exert numerous functions via binding to the glucocorticoid receptor (GR). The predominant active GC hormone is cortisol in humans, while rats and mice adrenals exclusively secrete corticosterone. The GR is a ligand-dependent transcription factor that is expressed in a wide variety of tissues and cell types (Weikum *et al.* 2017) and is activated during the circadian GC peak, upon stress, or due to hypercortisolism (de Kloet *et al.* 2005, Joëls 2018). The maintenance of homeostasis by GCs is tightly regulated by hypothalamus–pituitary–adrenal (HPA) axis activity (Lightman *et al.* 2020). The hypothalamic neurons from the paraventricular nucleus release the corticotropin-releasing hormone which reaches the corticotropic cells of the anterior pituitary gland and stimulates the release of adrenocorticotropic hormone (ACTH) into the circulation. ACTH in turn induces the production and secretion of GCs from the adrenal gland (Spencer & Deak 2017). Dysregulation of GC secretion is responsible for several pathologies, the most obvious being Cushing’s syndrome (CS). Endogenous CS is a condition characterized by cortisol overproduction that can be ACTH-dependent, caused by either a pituitary adenoma or an ectopic source, or ACTH-independent when caused by an adrenal gland tumor or hyperplasia (Barbot *et al.* 2020). Hypercortisolism in CS is associated with multisystem morbidity including all features of the metabolic syndrome, i.e. visceral fat accumulation, insulin resistance, hypertension and dyslipidemia (Kalsbeek *et al.* 2015), and with a range of neuropsychiatric symptoms such as depression, impaired memory and sleep disturbances (Pivonello *et al.* 2015). Similar symptoms are observed in some patients who use exogenous corticosteroid medication (Barbot *et al.* 2020).

Under normal physiological conditions, GCs exert negative feedback on the HPA axis to maintain homeostasis. This feedback mechanism is mediated by the GR via the hypothalamus and the hippocampus in the central nervous system (CNS), the pituitary and possibly the adrenal gland (Ratka *et al.* 1989, Walker *et al.* 2015). As such, GR agonists such as dexamethasone (DEX) suppress HPA axis activity and thereby diminish endogenous cortisol/corticosterone secretion (Andrews *et al.* 2012, Asirvatham *et al.* 2020). *Vice versa*, GR antagonists like mifepristone disinhibit HPA axis activity, resulting in increased ACTH production with concomitant elevated secretion of cortisol/corticosterone (Gaillard *et al.* 1984). GR antagonists counteract GC-driven metabolic disruption, but the disinhibition of the HPA axis can be an issue in such treatment regimens. The resulting elevated endogenous GCs compete for GR occupancy and can thereby interfere with effective GR antagonism. In addition, high GC levels can override the capacity of 11β-hydroxysteroid dehydrogenase type 2, that is expressed in the kidney and converts cortisol into inactive cortisone. This can lead to overactivation of mineralocorticoid receptors affecting the sodium/potassium balance (Ferrari 2010, Shearer *et al.* 2019). Unlike mifepristone, relacorilant selectively antagonizes GR activity while not affecting the progesterone receptor (PR) and the androgen receptor (AR) (Hunt *et al.* 2017). In phase 2 clinical trial, relacorilant was shown to be efficacious in reducing hyperglycemia, hypertension and other manifestations of hypercortisolism and was generally well-tolerated in patients with CS. Increases in ACTH and cortisol were substantially less than typically observed with mifepristone, which will have clinical advantages for patients with CS (Pivonello *et al.* 2021). Relacorilant is currently undergoing phase 3 clinical evaluation in this patient population (https://clinicaltrials.gov/ct2/show/NCT03697109). In the current preclinical study, we compared the effects of relacorilant and mifepristone *in vitro* and in male mice, with a focus on HPA axis (re-)activity.

## Methods

### Luciferase reporter assay in HEK-293 cells

HEK-293 cells were seeded as 80,000 cells/well in 24-well plates in culture medium DMEM (1X) + GlutaMAX™ with 10% charcoal-stripped FBS, supplemented with penicillin/streptomycin. The cells were transfected after 24 h using the FuGENE®HD Transfection Reagent (Promega Corporation) according to the manufacturer’s instructions. Per well, 10 ng human GR plasmid, 25 ng tyrosine aminotransferase (TAT1)-firefly-luciferase (reporter gene), 1 ng CMV-renilla-luciferase plasmid (control reporter gene) and 264 ng pcDNA were transfected. The TAT1 reporter construct is composed of a GR-responsive element of the *Tat* gene coupled to the firefly-luciferase and is transcribed upon GR activation (Liu *et al.* 1996, Kroon *et al.* 2021). One day later, we tested GR agonism using 0.1% dimethyl sulfoxide (DMSO) as vehicle, 1 µM relacorilant, 3 nM and 100 nM dexamethasone (DEX), or 100 nM and 1 µM cortisol. Both concentrations of DEX and cortisol corresponded to the EC_80_ and EC_max_ in HEK-293 cells. To test GR antagonism, cells were pre-treated for 1 h with 0.1–1000 nM relacorilant or mifepristone before treatment with either 3 nM DEX or 100 nM cortisol. After 24 h, the medium was removed, and the wells were washed once with PBS before the Dual-Luciferase® Reporter Assay (Promega Corporation) was performed. The luminescent signal was measured with SpectraMax®iD3, using SoftMax Pro 7.0.2.

### Human peripheral blood mononuclear cells

PBMCs were isolated from buffy coats of healthy donors by density gradient centrifugation using Ficoll Isopaque. PBMCs were seeded as 150,000 cells/well in RPMI medium supplemented with 10% charcoal-stripped FBS and penicillin–streptomycin. Cells were pretreated with 0.1–1000 nM relacorilant or mifepristone for 1 h before 100 nM DEX was added. After 24 h, cells were stimulated with 100 ng/mL lipopolysaccharide (LPS) for 6 h and supernatant was collected to determine human interleukin 6 (IL-6) levels by ELISA according to manufacturer’s protocol (Sanquin).

### Animals

#### Pharmacokinetics of relacorilant in vivo

Male CD-1 mice (Charles River) of 26–32 g were group-housed (*n* = 4/cage) in the Saretius animal facility at the University of Reading. Animals were maintained under 12 h light:12 h darkness cycle and were given *ad libitum* access to food and water. For i.v. dosing, mice were injected with 1 mg/kg relacorilant using a 5 mL/kg dosing volume in a vehicle consisting of 10% (v/v) DMSO and 90% (v/v) hydroxypropyl-β-cyclodextrin. i.v. dosing was facilitated by whole body warming in a hot box (Thermacage MK2, Datesand Ltd, Manchester, UK) set at approximately 40°C for not more than 10 min. For oral dosing, mice were administered with 5 mg/kg relacorilant using a 10 mL/kg dosing volume in a vehicle consisting of 10% (v/v) DMSO and 90% (v/v) hypromellose. Following dosing, mice were returned to their home cages. Three mice were killed by CO_2_ infixation at time points 0.083, 0.25, 0.5, 1, 2, 4, 8 and 24 h for i.v. dosing and 0.25, 0.5, 1, 2, 4, 6, 8 and 24 h for oral dosing. Blood samples were collected by cardiac puncture and were added to a 1.5 mL Eppendorf tube containing 5 μL of heparin (5000 IU/mL). The tubes were immediately sealed, mixed and centrifuged (Biofuge Pico, Kendro Lab Products, Osterode, Germany) at 9600 ***g*** for 3 min before transferring plasma samples (>100 μL) into fresh tubes. Samples were stored at −20°C until transport to the CEM Analytical Services Ltd. for LC-MS/MS measurements, using a turbo ion spray interface as the ion source and multiple reaction monitoring. A total of 10 µL acetonitrile: water (1:1) was added to 40 µL plasma. The samples were further diluted in 400 µL ice-cold acetonitrile, vortexed and centrifuged at 16,200 ***g*** for 5 min at 4°C. The supernatant was diluted in water (200 µL in 800 µL) in a HPLC vial. A total of 10 µL of the resulting samples were added tn a 50 × 4.6 mm C18 Ascentis Express 2.7 µm column with a mobile phase A (0.1% formic acid in water) and a mobile phase B (0.1% formic acid in acetonitrile) with an average flow rate of 1 mL/min. Data were then analyzed at Saretius Ltd. using Phoenix64/WinNonlin 6.3 and presented as mean. The plasma bioavailability (F) was calculated using the equation: F = (AUC_p.o__._/AUC_i.v_.) × (Dose_i.v__._/Dose_p.o__._) and expressed as a percentage (100 × F), where units are consistent for both AUCs (e.g. h × ng/mL) and for both doses (e.g. mg/kg). Brain concentrations of relacorilant in mice were assessed by administering 90 mg/kg relacorilant diluted in 10% DMSO, 0.1% Tween in hydroxypropyl methylcellulose (HPMC 5%) via oral gavage to six CD-1 male mice. Mice were killed by CO_2_ infixation 1 h (*n* = 3) or 3 h (*n* = 3) post-dosing. Brain samples were collected and homogenised in acetonitrile:water (1:1), with 300 µL for 100 mg. The resulting samples were added on a Acquity UPLC HSS T3 1.8 µm 2.1 × 50 mm column in the Xevo TQ-D UHPLC-MS/MS, using multiple reaction monitoring. The mobile phase A (0.1% formic acid) and mobile phase B (methanol) had an average flow rate of 0.6 mL/min.

#### Relacorilant treatment and continuous corticosterone exposure

The animal study was approved by the ethics committee of Leiden University Medical Center. Male 8-week-old C57BL/6J mice were housed in conventional cages with a 12 h light:12 h darkness cycle and were given *ad libitum* access to food and water. To evaluate relacorilant in a disease model of continuous glucocorticoid exposure, mice were subcutaneously implanted with 12.5 mg of corticosterone-release pellets (Koorneef *et al.* 2020, Kroon *et al.* 2021). Mice were treated via daily oral gavage (09:00 h) with 60 mg/kg relacorilant or vehicle (10% DMSO, 0.1% Tween-80, 0.5% hydroxypropylmethylcellulose in PBS). After 5 days, mice fasted for 6 h, and blood was collected for immune cell analysis (Sysmex XT-2000iV) and to analyze plasma biochemistry. Mice were killed by CO_2_ infixation, perfused with ice-cold PBS for 5 min and tissues were collected, snap-frozen in liquid nitrogen and stored at −80˚C until further processing.

#### Relacorilant treatment and acute dexamethasone exposure

The animal study was approved by the ethics committee of Leiden University Medical Center. Male 8-week-old C57BL/6J mice were housed in conventional cages with a 12 h light:12 h darkness cycle and were given *ad libitum* access to food and water. To test HPA axis responsiveness, a 3-day experiment was performed with daily administration of 60 mg/kg relacorilant, 60 mg/kg mifepristone or vehicle (10% DMSO, 0.1% Tween-80, 0.5% hydroxypropylmethylcellulose in PBS) via oral gavage (09:00 h). On day 1, a novelty stress test was performed 1 h after oral gavage treatment. Blood was collected at T = 0 min and animals were placed in a new cage without bedding for 10 min. Mice were then placed back in their home cages, and blood was collected at T = 30, 60, 120 and 180 min. On day 3, animals were subcutaneously injected with solvent (PBS) or 5 mg/kg of dexamethasone-phosphate for 1 h after the last oral gavage with vehicle, relacorilant or mifepristone. After 6 h, animals were killed by CO_2_ infixation, trunk blood was collected by heart puncture before the animals were perfused with ice-cold PBS for 5 min and tissues were collected, snap-frozen in liquid nitrogen and stored at −80˚C until further processing. Corticosterone levels were determined in all blood samples by using an HS EIA kit (Immunodiagnosticsystems, Boldon Business Park, UK).

### Plasma biochemistry

Plasma samples were analyzed for glucose using the Glucose liquiUV mono Multipurpose reagent (10786, Diagnostics Worldwide) for ELISA. The coefficient of variation (CV) for the glucose assay was 5.23% in commercial control serum (Autocal – IMTTC 13160, Diagnostics Worldwide) across 25 assays. Insulin was measured in plasma samples using the Ultra-Sensitive Mouse Insulin ELISA Kit (50-194-7920, Crystal Chem Inc.). According to the manufacturer’s instructions, the average CV was 25.82% in commercial control serum across 19 assays. The homeostatic model assessment for insulin resistance (HOMA-IR) was calculated by multiplying the fasted glucose concentration (mmol/L) by the fasted insulin concentration (mmol/L).

### RNA isolation, cDNA synthesis and RT-PCR analysis

Total RNA was isolated from liver, adrenal gland, pituitary gland, hippocampus, interscapular brown adipose tissue (iBAT) and gonadal white adipose tissue (gWAT) samples using TriPure Isolation reagent (Roche). We used 1 µg RNA for cDNA synthesis using the Recombinant RNasin® Ribonuclease Inhibitor kit (Promega Corporation) and BioRad T100™ Thermal Cycler. Gene expression was assessed by real-time quantitative PCR using GoTaq® RT-qPCR system (Promega Corporation) and thermal cycler Bio-Rad CFX96.

### Mouse AtT20 cells

Mouse corticotroph AtT20/D16v-F2 cells (ATCC number: CRL-1795) were routinely cultured in 75 cm^2^ flasks in DMEM containing 10% FCS, l-glutamine (2 mmol/L) and penicillin (100 U/mL) at 37°C in a humidified incubator at 5% CO_2_, as previously described (Creemers *et al.* 2021). Once every week, cells were harvested using trypsin (0.05%) – EDTA (0.48 mM) solution. For experiments, cells were seeded at a density of 40,000 cells/well in 24-well culture plates in 1 mL of culture medium. After 6 h, cells were treated with vehicle (0.1% EtOH or 0.1% DMSO), dexamethasone, relacorilant or mifepristone at indicated concentrations and combinations. After 72 h, supernatant was collected, protease inhibitor trasylol (5 IU/mL) was added to prevent ACTH degradation and samples were stored at −20°C. ACTH was measured using the Immulite 2000 XPi immunoassay analyzer (Siemens Medical Solutions). Cells were used for RNA isolation using a High Pure RNA kit (Roche, 11828665001) according to manufacturer’s protocol. All experiments were performed twice, and experimental groups were in quadruplicate. The relative expression of genes was normalized using the geometric mean of the expression of three reference genes (RG) *Hprt*, *GusB* and *Gapdh*, as previously described (Blažević *et al.* 2022).

### Microarray assay for real-time coregulator-nuclear receptor interaction

GR-coregulator interactions were assessed in the presence of 1 µM cortisol, mifepristone or relacorilant as compared to vehicle (DMSO), as previously described (Desmet *et al.* 2014). A total of 154 short leucine-rich binding motifs representing 64 known nuclear receptor coregulators were attached to the solid support of the PamChip® array. The coregulator-derived motifs were incubated with HEK-293 cell lysates containing a GR LGD-GST (Invitrogen), the ligand (DMSO, cortisol, mifepristone or relacorilant) and a tag-specific antibody coupled to a fluorophore. The interaction between GR and the coregulator motifs was assessed by detection of the immunofluorescent signal. For all coregulator motifs, each treatment condition had four replicates from which the mean and the standard error of mean (s.e.m) of the immunofluorescent signal were calculated and expressed as relative interaction. For cortisol, mifepristone and relacorilant treatments, the modulation index (MI) was calculated as the log_10_-transformed normalized interaction using DMSO as baseline.

### Statistical analysis

All data are expressed as mean (±s.e.m). Statistical significance was calculated using the one-way ANOVA or two-way ANOVA in GraphPad Prism 8. All normalized data were corrected for outliers using GraphPad outlier calculator. The IC50 values for relacorilant and mifepristone in HEK-293 and AtT20 cells, and DEX EC50 in AtT20 cells were determined using GraphPad Prism 8 non-linear fitting. The MI statistical significance was calculated using the t.test in R v.4.0.

## Results

### Relacorilant antagonizes GR signaling in HEK-293 cells

**Figure 1:**
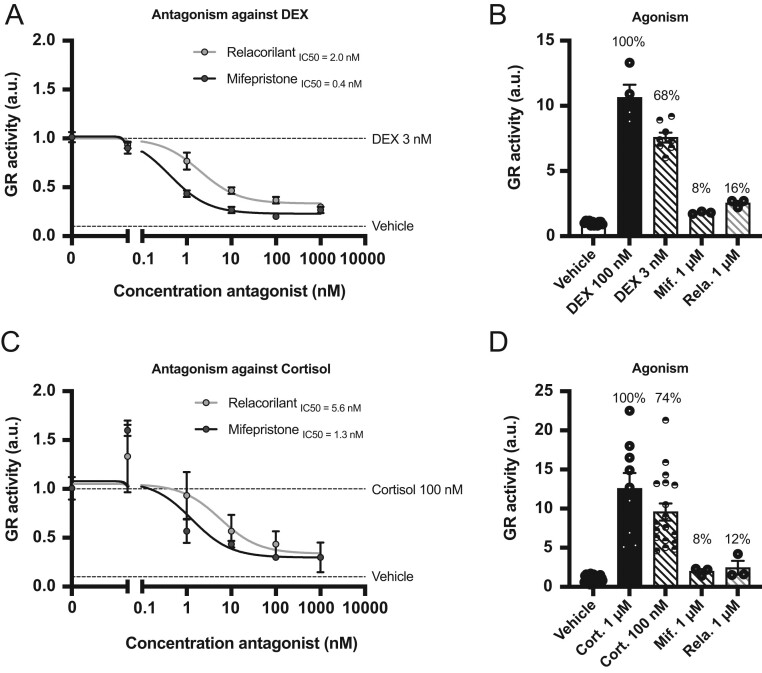
Relacorilant exhibits GR antagonism in human HEK-293 cells. GR activity measured via a luciferase reporter assay. (A) Antagonist mode with increasing doses of relacorilant or mifepristone competing with 3 nM DEX. (B) Agonist mode with 1 µM relacorilant or mifepristone as compared to 100 nM and 3 nM DEX. (C) Antagonist mode with increasing doses of relacorilant or mifepristone competing with 100 nM cortisol. (D) Agonist mode with 1 µM relacorilant or mifepristone as compared to 1 µM and 100 nM cortisol. All data are expressed as mean ±sem. The IC50 values were determined using GraphPad Prism 8 non-linear fitting. GR, glucocorticoid receptor; DEX, dexamethasone; Cort., cortisol; Mif., mifepristone; Rela., relacorilant.

HEK-293 cells were treated with increasing doses of relacorilant or mifepristone to evaluate antagonism of DEX- or cortisol-induced human GR signaling. Against 3 nM DEX, relacorilant showed a potency (IC50 = 2 nM) that was five-fold lower than mifepristone (IC50 = 0.4 nM) but reached the same efficacy at 1 µM (Fig. 1A). In agonism mode, both relacorilant and mifepristone were not significantly different as compared to the vehicle group (Fig. 1B). Against 100 nM cortisol, relacorilant effectively antagonized GR activity (IC50 = 5.6 nM), with a four-fold lower potency as compared to mifepristone (IC50 = 1.3 nM) (Fig. 1C). Consistent with previous findings, no significant GR agonism was observed for either relacorilant or mifepristone (Fig. 1D). The results suggest that relacorilant antagonizes GR activity in HEK-293 cells with a lower potency but a similar efficacy as mifepristone. Relacorilant binding does not result in any prominent GR agonism.

### Pharmacokinetic properties of relacorilant

We next evaluated relacorilant pharmacokinetics in CD-1 mice. Blood samples were collected via cardiac puncture at time points 0.083, 0.25, 0.5, 1, 2, 4, 8 and 24 h after i.v. injection of 1 mg/kg relacorilant and 0.25, 0.5, 1, 2, 4, 6, 8 and 24 h after oral administration of 5 mg/kg relacorilant. LC-MS/MS measurements of compound concentrations in the blood over time allowed the calculation of pharmacokinetic properties of relacorilant (Table 1).

**Table 1:** Pharmacokinetic properties of relacorilant following intravenous injection (i.v.; 1 mg/kg) or per os administration (p.o.; 5 mg/kg) in male CD-1 mice.

Parameters	Units	1 mg/kg i.v (mean)	5 mg/kg p.o (mean)
R^2^		0.9627	0.9805
C_m__ax_	ng/mL	623.9	130.0
T_m__ax_	h	0.083	0.5
AUC^(0-24)^	h × ng/mL	245.7	251.6
AUC^(0-∞)^	h × ng/mL	248.0	257.5
T_1/2_	h	0.69	1.62
Bioavailability (F)		20.5	

AUC(0-24), area under the concentration time curve to the last concentration measured; AUC(0-∞), area under the concentration time curve extrapolated to infinity based on the elimination rate constant Kel; C_max_, maximum concentration; F, bioavailability; R^2^, R squared, a measure of goodness of fit; T_max_, time point at which C_max_ occurs; T_1/2_, half-life of drug elimination.

The maximum detected concentration of relacorilant after i.v. dosing was 623.9 ng/mL at 0.083 h (5 min), with a plasma half-life of 0.69 h (41 min) following i.v. dosing. Upon oral dosing, the maximum concentration was 130 ng/mL at time point 0.5 h (30 min), with a plasma half-life of 1.62 h (97 min) following oral dosing. Based on these data, the bioavailability (F) of relacorilant was estimated at 20.5% (Table 1).

### Relacorilant treatment prevents glucocorticoid-induced hyperinsulinemia and immunosuppression

**Figure 2:**
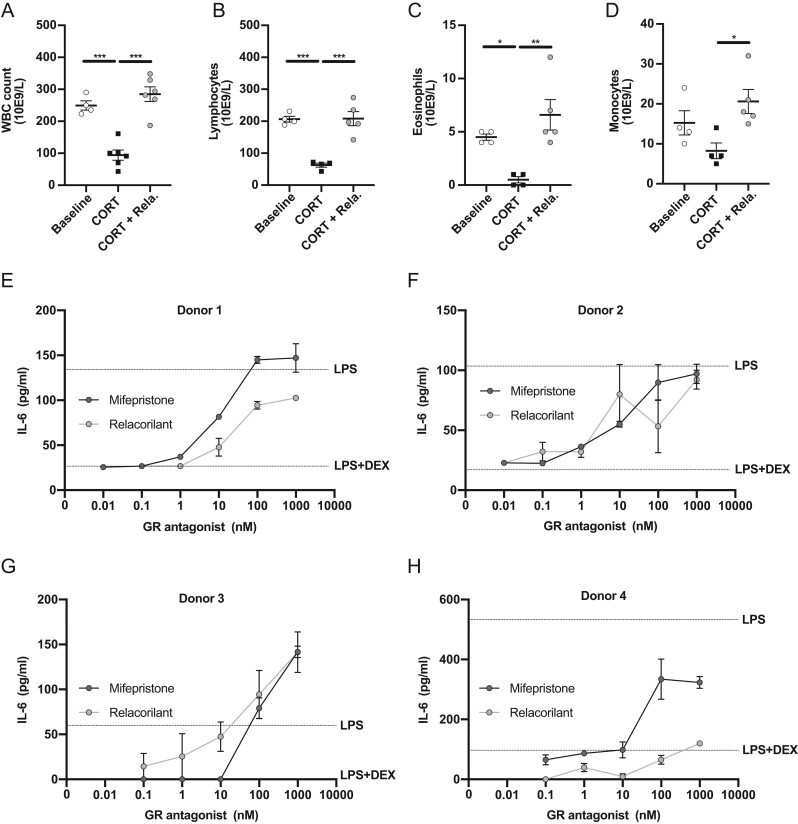
Relacorilant prevents glucocorticoid-induced immunosuppression in mice and anti-inflammatory effects in human PBMCs. The effect of 60 mg/kg relacorilant on corticosterone-induced reduction in (A) white blood cells, (B) lymphocytes, (C) eosinophils, (D) monocytes. (E-H) IL-6 release in 100 ng/mL LPS-stimulated human PBMCs from four donors after treatment with increasing doses of relacorilant or mifepristone competing with 100 nM DEX. All data are expressed as mean ± s.e.m. Statistical significance was calculated using GraphPad Prism 8 using a one-way ANOVA. WBC, white blood cells; CORT, corticosterone; Rela., relacorilant; IL-6, interleukin 6; LPS, lipopolysaccharide; DEX, dexamethasone.

We next tested the effects of relacorilant in mice, in a model of continuous corticosterone exposure (Koorneef *et al.* 2020, Kroon *et al.* 2021). Daily administration of relacorilant partially prevented corticosterone-induced hyperinsulinemia (*P* < 0.001, Supplementary Fig. 1A, see section on supplementary materials given at the end of this article) without influencing plasma glucose levels (Supplementary Fig. 1B), which resulted in a lower HOMA-IR (*P* < 0.01, Supplementary Fig. 1C). These beneficial effects of relacorilant on metabolic features were accompanied by effective GR antagonism on expression of its target genes *Fkbp5* and *Gilz* in metabolic tissues including liver, brown adipose tissue and white adipose tissue (Supplementary Fig. 1D, E and F). In addition to metabolic symptoms, continuous corticosterone exposure resulted in lower levels of total white blood cells (*P* < 0.001, Fig. 2A), lymphocytes (*P* < 0.001, Fig. 2B), eosinophils (*P* < 0.05, Fig. 2C) and monocytes (Fig. 2D), which were all normalized by relacorilant treatment (*P* < 0.001, Fig. 2A and B – *P* < 0.01, Fig. 2C– *P* < 0.05, Fig. 2D). To further investigate the effects of relacorilant on human immune cells, we utilized PBMCs isolated from four healthy donors in an *ex vivo* model of LPS-induced inflammation. DEX markedly reduced LPS-induced IL-6 production in all donors. Co-administration with mifepristone fully (donor 1–3) or partially (donor 4) prevented DEX-induced reduction of IL-6 levels (Fig. 2E, F, G and H). Co-administration with relacorilant partially prevented the effect of DEX in donor 1 (Fig. 2E), fully prevented the effect of DEX in donors 2 and 3 (Fig. 2F and G) and showed no effect in donor 4 (Fig. 2H). In conclusion, relacorilant treatment successfully counteracted corticosterone-induced hyperinsulinemia and immunosuppression in mice, as well as DEX-induced anti-inflammatory effects in human PBMCs for three out of four donors.

### Relacorilant treatment shows modest disinhibition of the HPA axis compared to mifepristone

**Figure 3:**
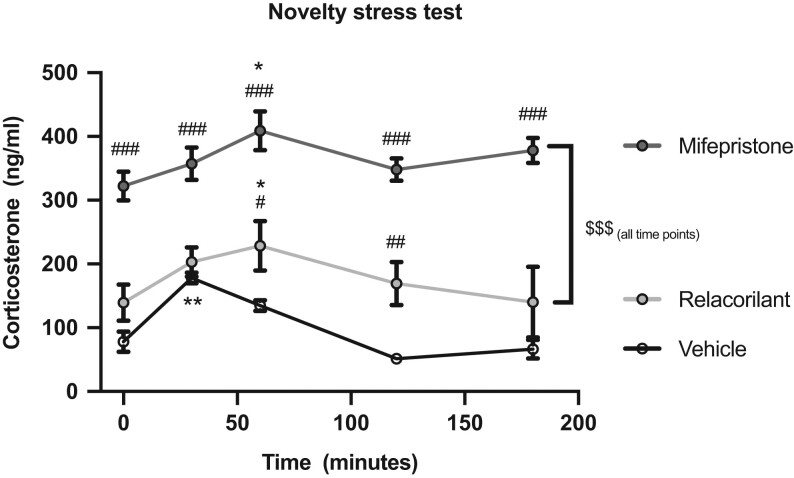
Relacorilant exhibits modest disinhibition of the HPA axis activity as compared to mifepristone. The effect of 60 mg/kg relacorilant and 60 mg/kg mifepristone on basal and novelty stress-induced plasma corticosterone levels. All data are expressed as mean ± s.e.m. Statistical significance was calculated using GraphPad Prism 8 using a two-way ANOVA. ^*^Significant difference with T = 0 within each treatment; ^#^Significant difference with vehicle at the same time point; ^$^Significant difference between mifepristone and relacorilant.

To evaluate the effects on HPA axis reactivity, a novelty stress test was performed in male mice 1 h after treatment with vehicle, mifepristone or relacorilant. In vehicle-treated mice, the novelty stressor significantly elevated plasma corticosterone levels at T = 30 min (*P* < 0.01 vs T = 0), and these gradually declined to reach pre-novelty stress levels at T = 120 min and T = 180 min (Fig. 3). Mifepristone pretreatment significantly increased plasma corticosterone before the novelty stressor (*P* < 0.001 vs vehicle). Corticosterone levels increased further due to the novelty stressor at T = 60 min (*P* < 0.01 vs T = 0) and remained high throughout the test (*P* < 0.001 vs vehicle at T = 30, 60, 120 and 180, Fig. 3). Relacorilant treatment significantly elevated corticosterone levels at T = 60 (*P* < 0.05) and T = 120 (*P* < 0.01) as compared to vehicle, demonstrating a relatively modest disinhibition of the HPA axis as compared to mifepristone (*P* < 0.001 between relacorilant and mifepristone at all time points). Novelty stress-induced corticosterone levels remained high at T = 180 after mifepristone but not relacorilant, suggesting rapid recovery of basal corticosterone levels upon relacorilant treatment (Fig. 3). In conclusion, relacorilant only modestly and transiently disinhibited the HPA axis, while mifepristone strongly disinhibited HPA axis activity, reflecting differences between the compounds with respect to interference with GR-mediated negative feedback.

### Relacorilant treatment antagonizes DEX-induced gene expression in the adrenal gland and the pituitary but not in the CNS

**Figure 4:**
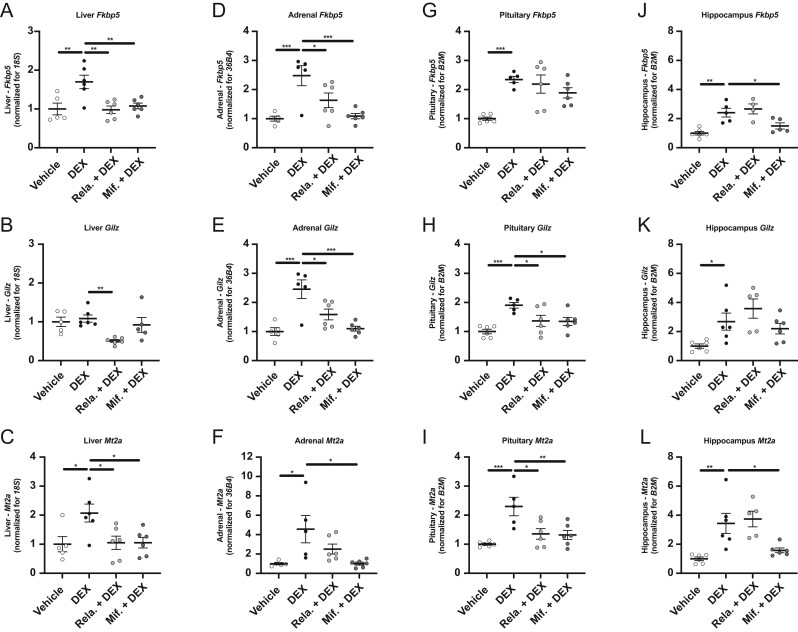
Relacorilant exhibits GR antagonism in peripheral tissues but not in the CNS. After 3 consecutive days of treatment with either vehicle, 60 mg/kg mifepristone or 60 mg/kg relacorilant, male mice were injected with 5 mg/kg DEX 1 h after the last treatment, and tissues were harvested 6 h later. The effect of relacorilant and mifepristone treatment on DEX-induced GR target gene expression (*Fkbp5*, *Gilz*, *Mt2a*) in mouse (A-C) liver, (D-F) adrenal gland, (G-I) pituitary and (J-L) hippocampus. All data are expressed as mean ± s.e.m. Statistical significance was calculated using GraphPad Prism 8 using a one-way ANOVA. DEX, dexamethasone; Mif., mifepristone; Rela., relacorilant; Fkbp5, FK506-binding protein 5; Gilz, glucocorticoid-induced leucine zipper protein; Mt2a, metallothionein 2A; 18S, 18S rRNA; 36B4, ribosomal phosphoprotein P0; B2M, beta-2-microglobulin.

Next, we more directly assessed GR antagonism *in vivo*. After 3 consecutive days of treatment with either vehicle, mifepristone or relacorilant, male mice were injected with 5 mg/kg DEX 1 h after the last treatment, and tissues were harvested 6 h later. DEX treatment completely suppressed HPA axis activity as apparent from diminished endogenous corticosterone levels (Supplementary Fig. 2A). This effect was neither influenced by relacorilant nor mifepristone pre-treatment (Supplementary Fig. 2A). Negative feedback on the HPA axis is regulated at multiple levels* i.e.* in the adrenal gland, the pituitary, the hypothalamus and the hippocampus. To evaluate GR antagonism at different levels of the HPA axis, we performed RT-qPCR analysis of well-known GR-responsive genes *Fkbp5*, *Gilz* and* Mt2a*. As a positive control for peripheral antagonism of GR signaling, we included gene expression analysis in the liver. In this tissue, relacorilant and mifepristone both effectively reduced DEX-induced *Fkbp5* and *Mt2a* expression (*P* < 0.01 and *P* < 0.05 respectively, Fig. 4A and 4C), while *Gilz* expression was lowered by relacorilant but not by mifepristone treatment (*P* < 0.01, Fig. 4B). In the adrenal gland, DEX-induced upregulation of *Fkbp5* and *Gilz* was prevented by both relacorilant (*P* < 0.05) and mifepristone (*P* < 0.001) (Fig. 4D and E), while DEX-induced increase of *Mt2a* expression was only significantly reduced by mifepristone (*P* < 0.05, Fig. 4F). In the pituitary, relacorilant and mifepristone did not lower *Fkbp5* expression (Fig. 4G) but significantly lowered DEX-induced *Gilz* (*P* < 0.05, Fig. 4H) and *Mt2a* expression (*P* < 0.05 and *P* < 0.01 respectively, Fig. 4I). As a proxy for GR signaling in the CNS, we analyzed gene expression in the hippocampus, where mifepristone significantly reduced DEX-induced expression of *Fkbp5* (*P* < 0.05) and *Mt2a* (*P* < 0.05) but not *Gilz* (Fig. 4J, K and L). Relacorilant, on the other hand, did not influence any DEX-induced GR target gene expression in the hippocampus (Fig. 4J, K and L). We next investigated the expression of genes with tissue-specific function. Hepatic *Mttp* and adrenal *Cyp11b1* were not influenced by DEX or GR antagonist pre-treatment (Supplementary Fig. 2B and C). In the pituitary, expression of *Pomc* (precursor for ACTH) was downregulated by DEX but not influenced by relacorilant nor mifepristone (Supplementary Fig. 2D). Hippocampal *Sgk1* was upregulated after DEX injection but neither influenced by relacorilant nor mifepristone treatment (Supplementary Fig. 2E). Finally, *Nr3c1* (*Gr*) expression was altered by DEX treatment in the pituitary but not in the other tissues of interest (*P* < 0.001, Supplementary Fig. 2F, G, H and I), and this effect was not further influenced by either relacorilant or mifepristone pre-treatment (Supplementary Fig. 2H). In the adrenal gland, *Nr3c1* expression was higher after relacorilant treatment as compared to mifepristone (*P* < 0.01, Supplementary Fig. 2G). Altogether, the results suggest that relacorilant exerts GR antagonism in peripheral tissues but not in the CNS.

### Relacorilant is less potent than mifepristone in AtT20 mouse pituitary cells

**Figure 5:**
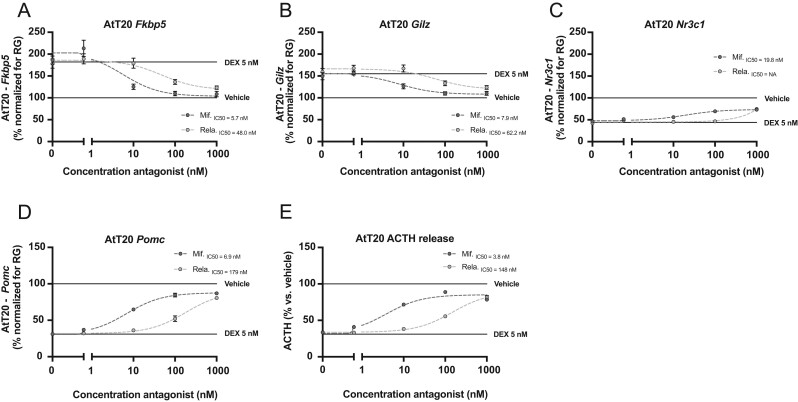
Relacorilant exhibits GR antagonism in mouse pituitary AtT20 cells. The effect of different doses of relacorilant or mifepristone on the effects of 5 nM DEX on (A) *Fkbp5*, (B) *Gilz*, (C) *Nr3c1*, (D) *Pomc* expression, and (E) ACTH release. All data are expressed as mean ± s.e.m. The IC50 values were determined using GraphPad Prism 8 non-linear fitting. The relative expression of genes was normalized using the geometric mean of the expression of three reference genes (RG) *Hprt*, *GusB* and *Gapdh*. DEX, dexamethasone; Mif., mifepristone; Rela., relacorilant; Fkbp5, FK506-binding protein 5; Gilz, glucocorticoid-induced leucine zipper protein; Nr3c1, nuclear receptor subfamily 3 group C member 1; Pomc, proopiomelanocortin; ACTH, adrenocorticotropic hormone; Hprt, hypoxanthine phosphoribosyltransferase 1; GusB, glucuronidase beta; Gapdh, glyceraldehyde-3-phosphate dehydrogenase.

Slow feedback on the HPA axis by synthetic glucocorticoids takes place at the level of GR-mediated gene expression in the anterior pituitary. In addition, pituitary tumors constitute a common cause of endogenous CS (Barbot *et al.* 2020). We therefore evaluated the effects of relacorilant on DEX-induced gene expression in the mouse AtT20 pituitary cell line, in comparison with mifepristone. AtT20 cells were treated with vehicle or 1 nM – 1 µM relacorilant or mifepristone in the presence of 5 nM DEX (corresponding to the EC80 for the measured genes, Supplementary Fig. 3A). DEX induced the expression of *Fkbp5* and *Gilz* *,* which was dose-dependently prevented by relacorilant and mifepristone treatment, and IC50 values of relacorilant were approximately eight times higher as compared to mifepristone (Fig. 5A and B). Both relacorilant and mifepristone only partially prevented DEX-induced downregulation of *Nr3c1* expression (Fig. 5C). Finally, DEX treatment suppressed *Pomc* expression and ACTH release, which was prevented by both GR antagonists. Relacorilant showed a substantially lower potency as compared to mifepristone with a 26-fold difference in IC50 for *Pomc* expression and a 38-fold difference for ACTH release (Fig. 5D and E). Altogether, these results suggested that both relacorilant and mifepristone antagonized GR signaling in mouse AtT20 pituitary cells, but relacorilant showed lower potency as compared to mifepristone, in particular with respect to ACTH production and release. These results demonstrate that the difference in potency between mifepristone and relacorilant depends on a GR-dependent process and was most substantial for the suppression of *Pomc* transcription and ACTH release.

### Relacorilant induces a unique GR interactome that lacks two key transcriptional corepressors

**Figure 6:**
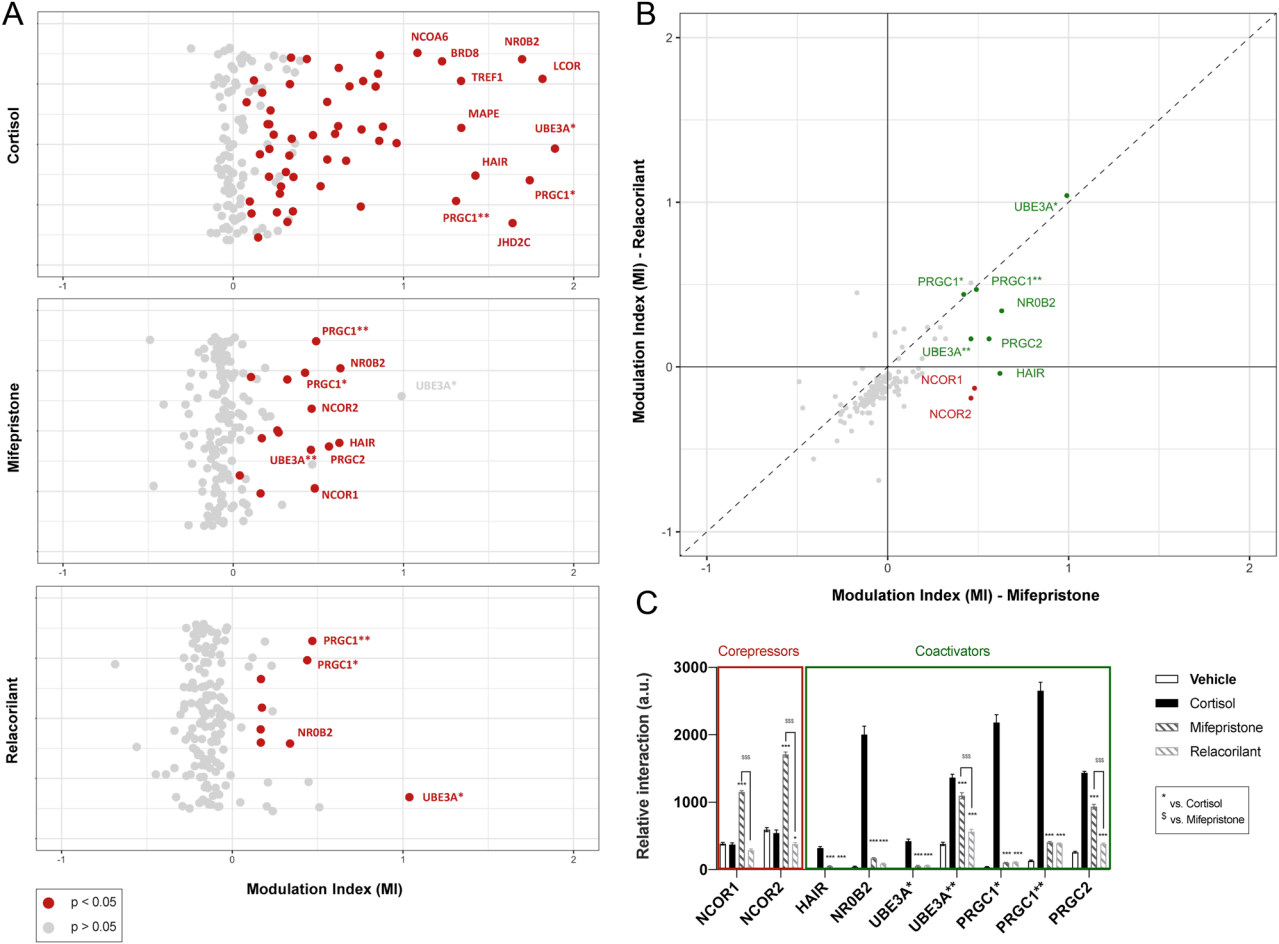
Relacorilant induces a unique GR interactome. (A) Interaction score of GR with 154 short leucine-rich binding motifs of 64 known nuclear receptor coregulators as measured by the modulation index (MI) in the presence of 1 µM cortisol, mifepristone or relacorilant. (B) Fold change-fold change plot of the interaction score of GR with nuclear receptor coregulator motifs as measured by the modulation index (MI) in the presence of 1 µM relacorilant compared to 1 µM mifepristone. (C) Interaction score of GR with a subset of nuclear receptor coregulator motifs as measured by relative binding in the presence of 1 µM cortisol, mifepristone or relacorilant. The relative interaction is expressed as mean ± s.e.m. and statistical significance was calculated using a one-way ANOVA in GraphPad Prism 8. ^*^Significant difference with cortisol; ^$^Significant difference between mifepristone and relacorilant. The MI is expressed as the log_10_-transformed normalized relative interaction, and statistical significance was calculated with *t-*test using R v.4.0.

The proteins that form the transcriptional complexes that mediate GR-dependent gene transcription differ per cell type and per gene. In view of the gene-dependent potency of relacorilant and mifepristone, we next characterized the GR interactome in the presence of 1 µM cortisol, mifepristone or relacorilant. We assessed the interaction between the ligand-binding domain of GR and 154 short leucine-rich binding motifs representing 64 known nuclear receptor coregulators. GR antagonists typically only stimulate a limited subset of GR interactions with transcriptional coregulators (Zalachoras *et al.* 2013, Viho *et al.* 2019). In the presence of cortisol, GR showed a significant interaction with a total of 55 motifs, while mifepristone and relacorilant significantly induced GR interaction with 15 and 8 motifs, respectively (*P* < 0.05, Fig. 6A). As anticipated, we also observed similar interaction scores for many GR coactivators for both GR antagonists (Fig. 6B). Several GR interactions were substantially weaker in the presence of GR antagonists as compared to cortisol for example, HAIR, NR0B2, PRGC1 and PRGC2 (*P* < 0.001, Fig. 6C, Supplementary Fig. 3B). When focusing further on the differences in the GR recruitment profile of both GR antagonists, we observed that relacorilant did not result in GR recruitment of corepressor motifs NCOR1 and NCOR2, while these were readily recruited by GR in the presence of mifepristone (*P* < 0.001, Fig. 6B and C, Supplementary Fig. 3B). In fact, NCOR1 and NCOR2 were specific to the mifepristone-bound GR complex as these interactions were also lacking in the presence of cortisol (*P* < 0.001, Fig. 6C, Supplementary Fig. 3B). Our results suggested that both mifepristone and relacorilant induced an antagonist-like GR transcriptional interactome, but the antagonistic properties of mifepristone seemed to involve GR interaction with corepressors such as NCOR1 and NCOR2, while relacorilant antagonism seemed to depend exclusively on the absence of coactivators in the GR interactome.

## Discussion

**Figure 7:**
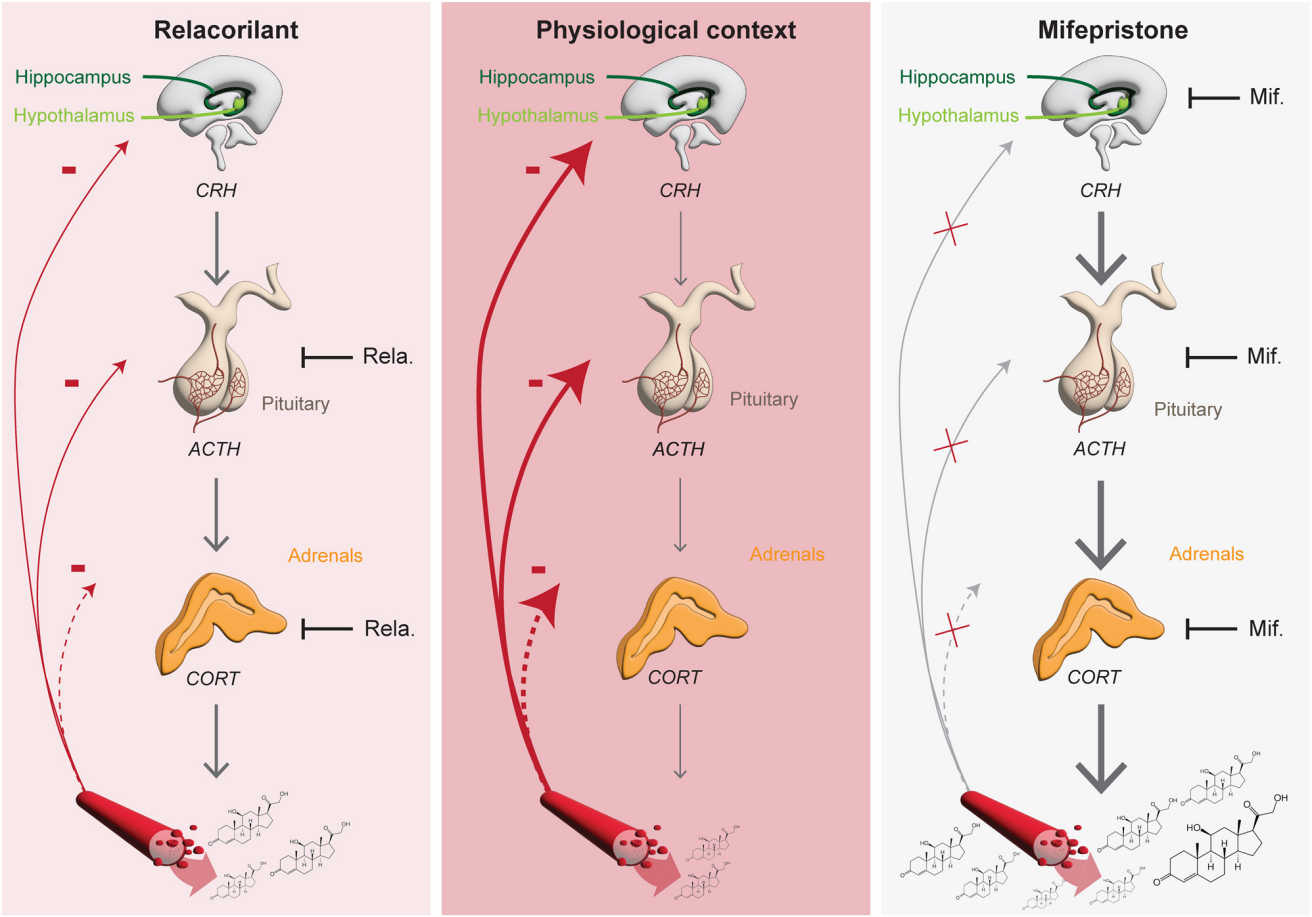
Graphical overview of the effects of relacorilant and mifepristone on HPA axis activity. Under normal physiological conditions in mice (central panel), CRH is released from the hypothalamus and stimulates the release of ACTH from the pituitary gland. ACTH induces the secretion of corticosterone from the adrenal gland into the bloodstream. Corticosterone exerts – via the GR – a negative feedback on the HPA axis to maintain homeostasis. Mifepristone treatment antagonizes the GR in the CNS, the pituitary and the adrenal gland which results in a strong disinhibition of the HPA axis and a significant increase of endogenous ACTH and corticosterone (right panel). Relacorilant treatment exhibits GR antagonism in the pituitary and the adrenal gland but to a much lesser extent in the CNS. Therefore, GR antagonism by relacorilant only results in a modest and transient disinhibition of the HPA axis which limits the increase of endogenous ACTH and corticosterone (left panel). CRH, corticotropin-releasing hormone; ACTH, adrenocorticotropic hormone; CORT, corticosterone; Mif., mifepristone; Rela., relacorilant.

In this study, we set out to further characterize the selective GR antagonist relacorilant preclinically, in particular with respect to HPA axis disinhibition (Fig. 7). There is a great need for improved GR antagonists, as the only marketed GR antagonist, mifepristone, exhibits cross-reactivity for the PR and is clinically associated with numerous side effects such as irregular vaginal bleeding and endometrial thickening (Sun *et al.* 2014). The use of mifepristone is also associated with mineralocorticoid effects (hypokalemia, hypertension and edema) (Yuen *et al.* 2015, Sai *et al.* 2019, Brown *et al.* 2020). Effective GR antagonism with relacorilant was previously shown in healthy human subjects, in which single or multiple daily dosing of relacorilant was reported to reduce prednisone-induced *Fkbp5* expression in blood (Bali *et al.* 2016) and reversed the decrease in eosinophils caused by prednisone treatment (Hunt *et al.* 2018). In mice, we observed additional beneficial effects as relacorilant co-administration prevented corticosterone-induced hyperinsulinemia and immunosuppression. Due to the short length of this experiment, we were unable to evaluate the full range of metabolic effects of relacorilant treatment in this cohort. Our current results also confirmed GR antagonism in human HEK-293 cells and mouse AtT20 cells and showed that relacorilant treatment prevented the anti-inflammatory effect of DEX in human PBMCs exposed to LPS.

Mifepristone treatment causes disinhibition of the HPA axis by inhibiting negative feedback, and this results in excessive levels of circulating ACTH and cortisol (Morgan & Laufgraben 2013). Previous results in patients with endogenous CS due to pituitary corticotroph adenomas showed that long-term treatment with mifepristone resulted in a continuous elevation of circulating ACTH (Fleseriu *et al.* 2014), driving subsequent cortisol elevations. In the same patient population, treatment with relacorilant was not associated with a significant increase in serum ACTH and cortisol concentrations (Pivonello *et al.* 2021). In a novelty stress test in mice, we confirmed the strong disinhibition of the HPA axis by mifepristone treatment (Gaillard *et al.* 1984, Ratka *et al.* 1989). In contrast, relacorilant treatment only modestly and transiently disinhibited the HPA axis in mice *in vivo*. This was in line with our *in vitro* observations in mouse corticotroph cells in which relacorilant less potently counteracted DEX-mediated suppression of *Pomc* mRNA and ACTH release compared to mifepristone. Interestingly, the fold difference between relacorilant and mifepristone for suppression of *Pomc* mRNA and ACTH release was four times larger in comparison to the effect on classical GR target genes *Fkbp5* and *Gilz*, and *Nr3c1* (GR). Thus, there seems to be an intrinsically low potency of relacorilant in the regulation of components of the HPA axis. It is important to note that AtT20 cells differ from normal corticotroph cells. For example, they have dysfunctional arginine-vasopressin (AVP) receptors, as AVP treatment fails to alter ACTH secretion (Lutz-Bucher *et al.* 1987). Therefore, differential modulation of ACTH secretion by the two GR antagonists may not reflect the *in vivo* situation.

Many GC effects are genomic and mediated by intracellular GRs; however, rapid effects on ACTH secretion can also be mediated by non-genomic pathways (Uchoa *et al.* 2014). It was previously shown in AtT20 cells and rat primary pituitary cells that corticosterone-mediated inhibition of ACTH secretion involved membrane-associated GRs (Deng *et al.* 2015). Although mifepristone is unable to inhibit rapid GC effects in rat hypothalamus (Di *et al.* 2003), it is possible that mifepristone acts via membrane-associated GRs in corticotroph cells to a different extent than relacorilant. It is also clear from the study of GR^dim^ ^/dim^ mice that ACTH production and release exhibit different sensitivity to GR-mediated inhibition (Reichardt *et al.* 1998). In these mice with impaired genomic GR signaling, *Pomc* mRNA levels were clearly altered via a genomic mechanism, but ACTH blood levels were not strongly affected. In the current study, the difference in potency of the two GR antagonists may therefore reflect differences in both genomic and non-genomic regulatory mechanisms.

Differences between relacorilant and mifepristone treatment could be the result of different pharmacokinetic properties. The pharmacokinetics of mifepristone have been assessed in rats, monkeys and humans but not in mice. It has been reported to be metabolized within 1–4 h following oral administration in rats with a bioavailability (F) of 39% (Deraedt *et al.* 1985, Sitruk-Ware & Spitz 2003), and within 20–30 h in humans (Heikinheimo 1997, Sarkar 2002). In mice, relacorilant had a plasma half-life of 1.62 h (97 min) following oral dosing with a bioavailability (F) of 20.5% (Table 1). Based on these data, we estimate that the half-life of mifepristone is slightly longer than relacorilant. There are major differences in half-life between rodents and humans, as in humans, the half-life of relacorilant is between 11 and 19 h (Hunt *et al.* 2018). Relacorilant treatment in humans results in lower ACTH and cortisol elevations as compared to mifepristone (Fleseriu *et al.* 2012, 2014, Pivonello *et al.* 2021), which we interpret as lower HPA axis disinhibition. It seems unlikely that species differences in plasma pharmacokinetics explain the observed functional differences.

Relacorilant and mifepristone diverge in pharmacodynamic terms of GR transcriptional coregulator recruitment *in vitro*. It was previously shown that the extent of antagonism on GR-mediated gene expression can depend on transcriptional cofactor availability and ratios, which can differ in certain tissues or cell types (Meijer *et al.* 2005, Mahfouz *et al.* 2016, Viho *et al.* 2019, 2022, Koorneef *et al.* 2020). Our results suggest that the antagonistic properties of mifepristone on the GR may rely on interaction with transcriptional corepressors NCOR1 and NCOR2, while GR antagonism by relacorilant seems to be associated with less coactivator recruitment in general. However, we have no direct evidence of the causal effects of GR interactome on the differential effects of relacorilant and mifepristone. Differential coregulator recruitment may also underly the gene-dependent differences in IC50 values between HEK-293 and AtT20 cells. The reduced potency of relacorilant in AtT20 cells was most pronounced for the suppression of *Pomc* mRNA and ACTH release but extended to generic GR target genes such as *Fkbp5*. Therefore, in addition to a gene-specific phenomenon, there could be a cell type-specific component to the potency of the two GR antagonists, analogous to our earlier observations for the GR antagonist CORT125281 in adipose tissue (Koorneef *et al.* 2020).

Another possible explanation of these differential effects of mifepristone and relacorilant on HPA axis disinhibition is the lower degree of GR antagonism by relacorilant in the CNS. In CD-1 mice, relacorilant was readily detected in the brain (Supplementary Fig. 3C). Assuming that brain penetration is not mouse-strain specific, we expect brain penetrance of relacorilant for the two C57BL/6J mouse cohorts used in the current study. Brain penetrance of relacorilant in humans has not been directly assessed, but is assumed, based on improvement of psychiatric symptoms in patients with CS treated with relacorilant (Pivonello *et al.* 2021). Differences in brain penetration cannot explain the lesser elevations in endogenous GCs that are observed in mice and patients treated with relacorilant, as compared to mifepristone. Since relacorilant reaches the brain, we must again evoke the argument of a specific GR interactome induced by relacorilant to explain its lack of GR antagonism on hippocampal gene expression.

In conclusion, the current study shows that the selective GR antagonist relacorilant only modestly affects HPA axis activity, which may provide substantial clinical benefit for CS patients as compared to mifepristone. Relacorilant treatment exhibits GR antagonism in the pituitary and the adrenal gland but to a much lesser extent in the CNS. Therefore, GR antagonism by relacorilant only results in a modest and transient disinhibition of the HPA axis which limits the increase of endogenous ACTH and corticosterone (Fig. 7). These distinct properties of relacorilant possibly rely on a unique ligand-induced GR interactome which differentially affects GR biology in the CNS and the periphery.

## Supplementary Material

Supplementary Figure 1. The effect of relacorilant pre-treatment (60 mg/kg via daily oral gavage) on corticosterone-induced (A) plasma insulin, (B) plasma glucose, and (C) the HOMA-IR index. The effect of relacorilant on mRNA expression in (D) the liver, (E) iBAT, and (F) gWAT. All data are expressed as mean ±sem. Statistical significance was calculated using GraphPad Prism 8 using a one-way ANOVA. Abbreviations: CORT = corticosterone; Rela. = relacorilant; HOMA-IR = Homeostatic Model Assessment for Insulin Resistance; Fkbp5 = FK506 binding protein 5; Gilz = Glucocorticoid-Induced Leucine Zipper Protein; Mttp = Microsomal Triglyceride Transfer Protein; Ucp1 = Uncoupling Protein 1; Atgl = Adipose Triglyceride Lipase; 18S = 18S ribosomal RNA.

Supplementary Figure 2. (A) The effect of 60 mg/kg relacorilant or 60 mg/kg mifepristone on plasma corticosterone levels after DEX injection. The effect of relacorilant and mifepristone treatment on DEX-induced expression of (B) liver Mttp expression, (C) adrenal gland Cyp11b1 expression, (D) pituitary Pomc expression and (E) hippocampus Sgk1 expression. Nr3c1 expression in (F) the liver, (G) the adrenal gland, (H) the pituitary, and (I) the hippocampus. All data are expressed as mean ±sem. Statistical significance was calculated using GraphPad Prism 8 using a one-way ANOVA. Abbreviations: DEX = dexamethasone; Mif. = mifepristone; Rela. = relacorilant; HOMA-IR = Homeostatic Model Assessment for Insulin Resistance; Fkbp5 = FK506 binding protein 5; Gilz = Glucocorticoid-Induced Leucine Zipper Protein; Mttp = Microsomal Triglyceride Transfer Protein; Cyp11b1 = Cytochrome P450 Family 11 Subfamily B Member 1; Pomc = Proopiomelanocortin; Sgk1 = Serine/threonine-protein kinase; Nr3c1 = Nuclear Receptor Subfamily 3 Group C Member 1; 18S = 18S ribosomal RNA; 36B4 = ribosomal phosphoprotein P0; B2M = Beta-2-Microglobulin.

Supplementary Figure 3. (A) DEX dose-response curve for Fkbp5, Gilz, Pomc and Nr3c1 in mouse AtT20 pituitary cells. All data are expressed as mean ±sem. The EC50 values were determined using GraphPad Prism 8 non-linear fitting. (B) Fold change-fold change plot of the interaction score of GR with nuclear receptor coregulator motifs as measured by the modulation index (MI) in the presence of 1 µM mifepristone or 1 µM relacorilant compared to 1 µM cortisol. (C) Relacorilant brain concentrations in CD-1 mice one and three hours after administration of 90 mg/kg relacorilant via oral gavage. All data are expressed as mean ±sem. Abbreviations: DEX = dexamethasone; Fkbp5 = FK506 binding protein 5; Gilz = Glucocorticoid-Induced Leucine Zipper Protein; Pomc = Proopiomelanocortin; Nr3c1 = Nuclear Receptor Subfamily 3 Group C Member 1.

Supplementary table 1. Detailed sequences of all qPCR primers.

## Declaration of interest

HJ Hunt is an employee of Corcept Therapeutics, who develops Relacorilant. J Kroon is seconded to Corcept Therapeutics. Corcept Therapeutics provides funding to OC Meijer.

## Funding

This study was funded by Corcept Therapeutics.

## Author contribution statement

EMG Viho and J Kroon contributed equally to the study.
