# Supplementary figures and images for: Peripheral glucocorticoid receptor antagonism by relacorilant with modest HPA axis disinhibition
Source: J Endocrinol. 2022 Dec 22;256(2):e220263. doi: 10.1530/JOE-22-0263 (PMC9874980; doi:10.1530/JOE-22-0263)

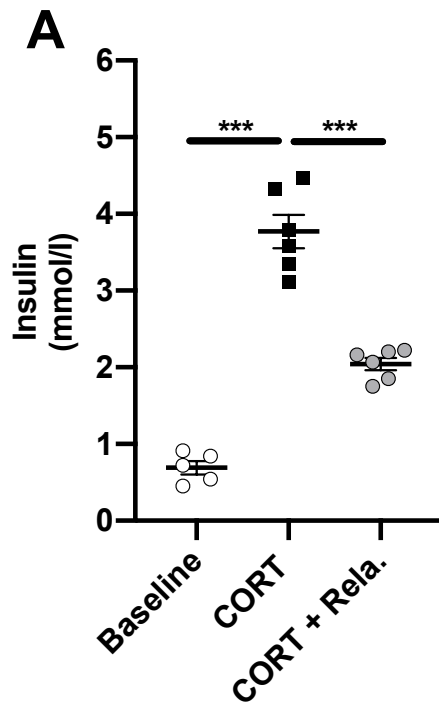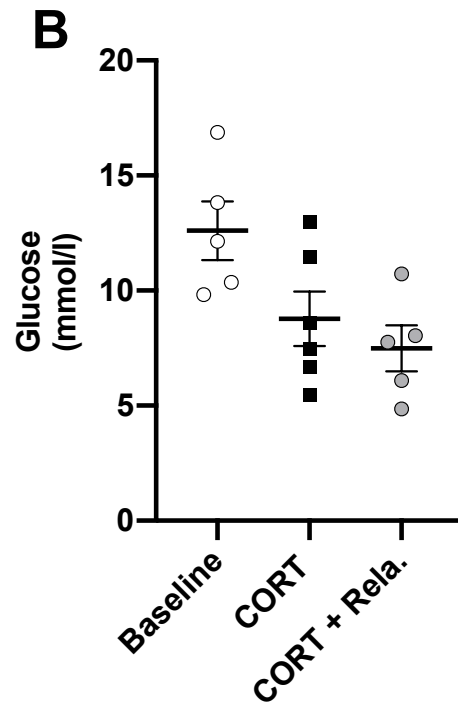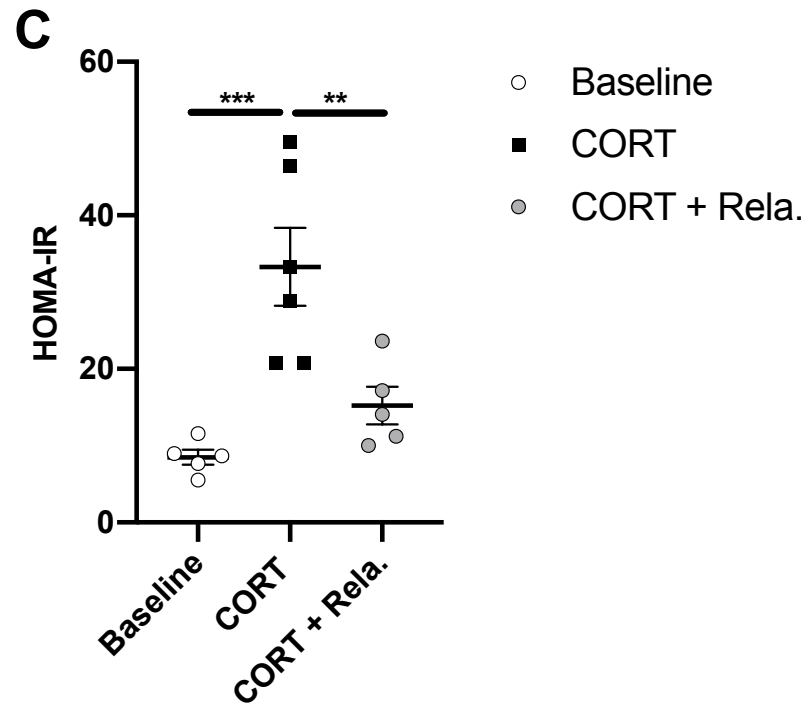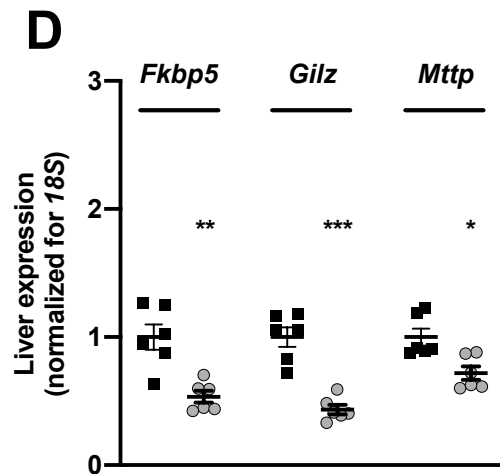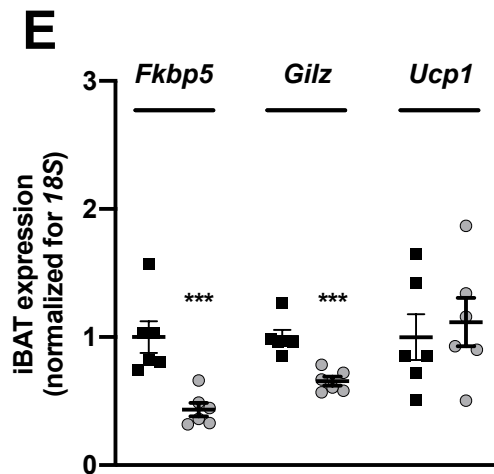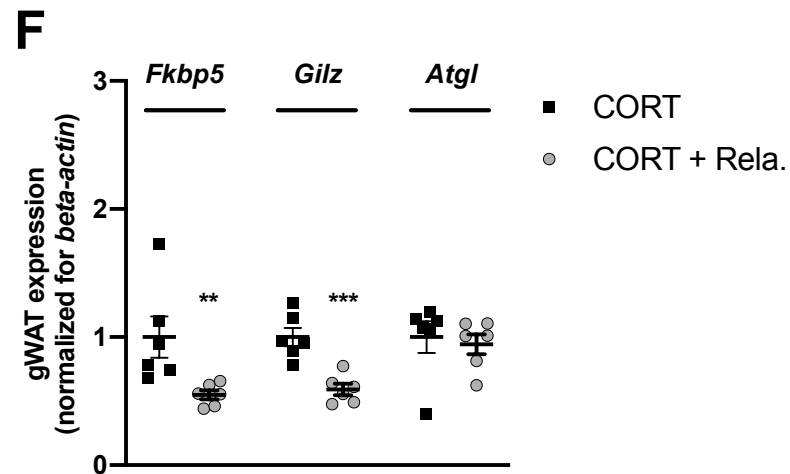

Supplement: Supplementary Figure 1. The effect of relacorilant pre-treatment (60 mg/kg via daily oral gavage) on corticosterone-induced (A) plasma insulin, (B) plasma glucose, and (C) the HOMA-IR index. The effect of relacorilant on mRNA expression in (D) the liver, (E) iBAT, and (F) gWAT. All data are expresse [file supplementary_figure_1.pdf]

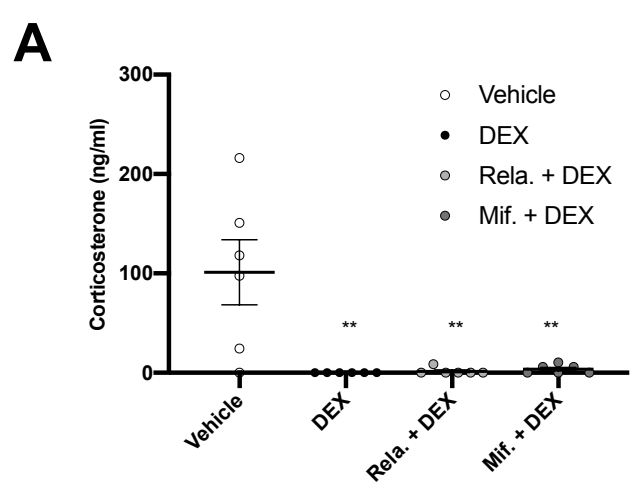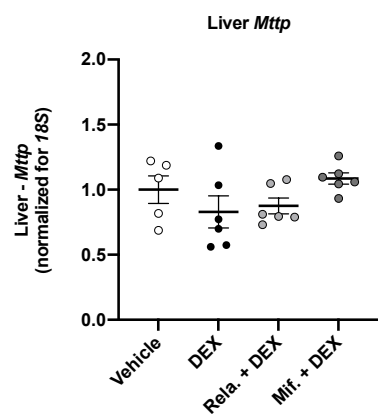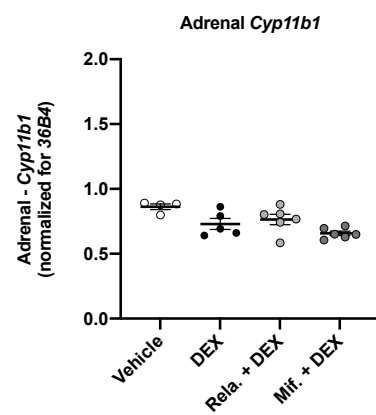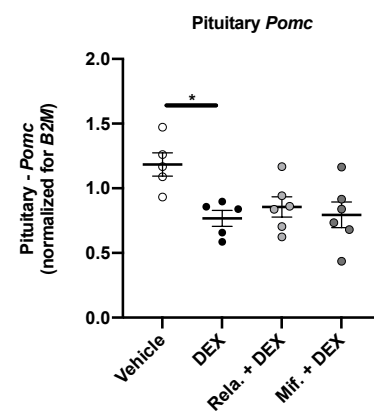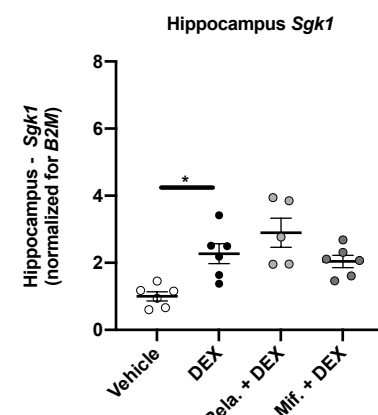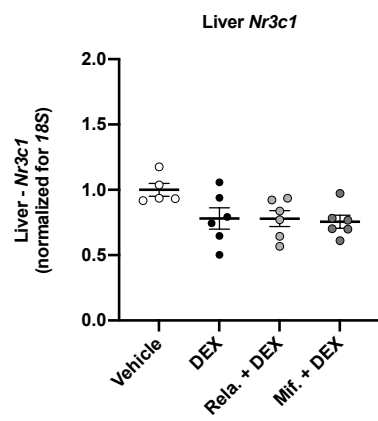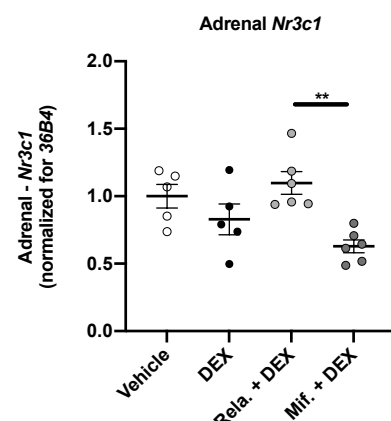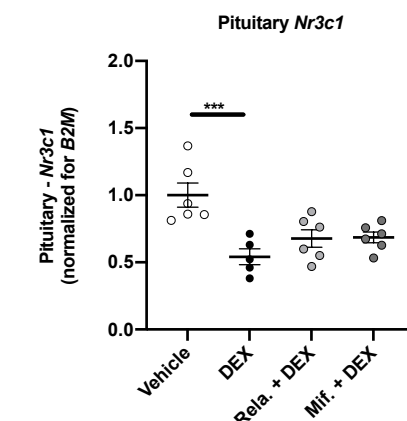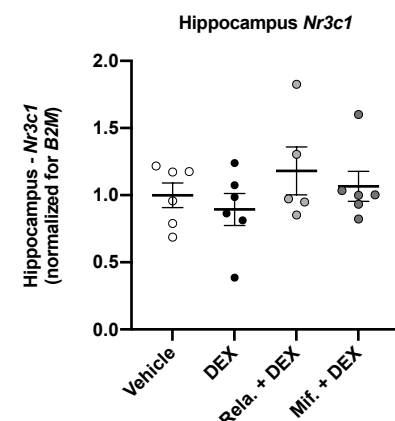

Supplement: Supplementary Figure 2. (A) The effect of 60 mg/kg relacorilant or 60 mg/kg mifepristone on plasma corticosterone levels after DEX injection. The effect of relacorilant and mifepristone treatment on DEX-induced expression of (B) liver Mttp expression, (C) adrenal gland Cyp11b1 expression, (D) pituit [file supplementary_figure_2.pdf]

A

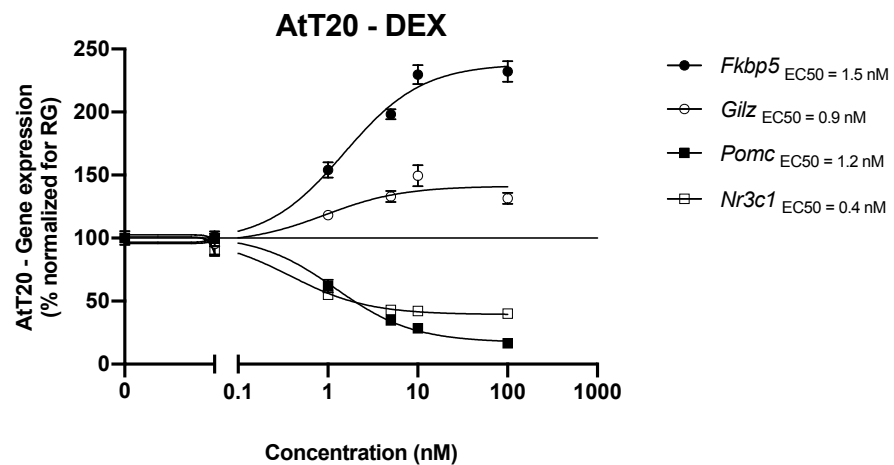

C

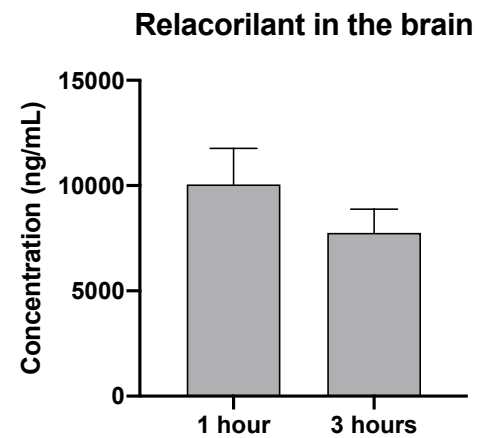

B

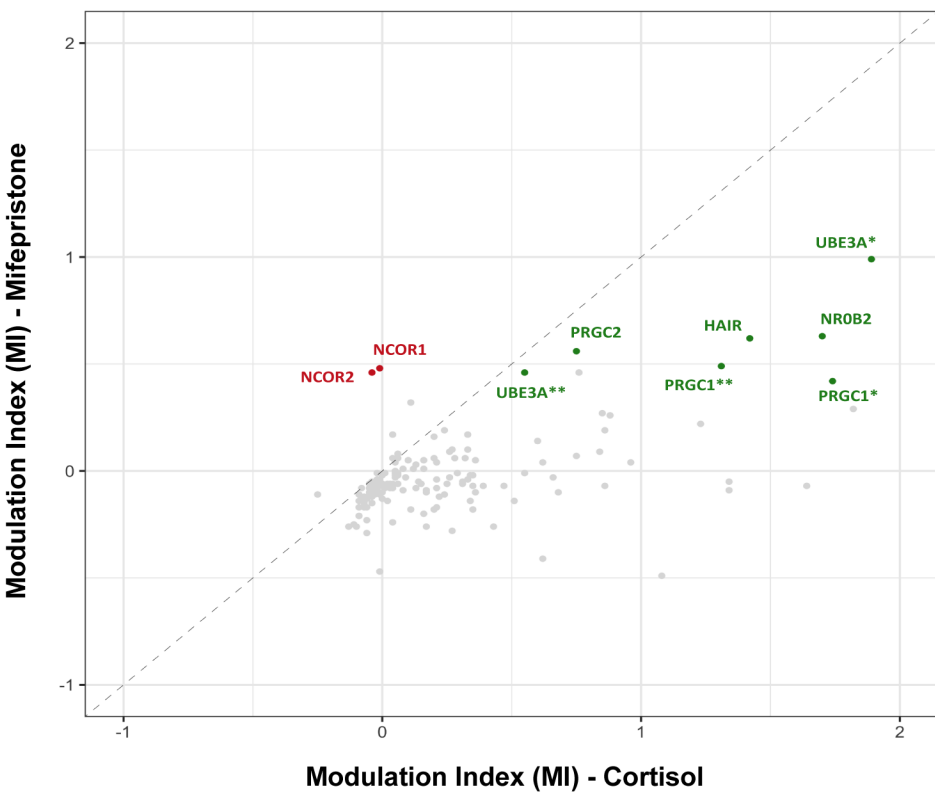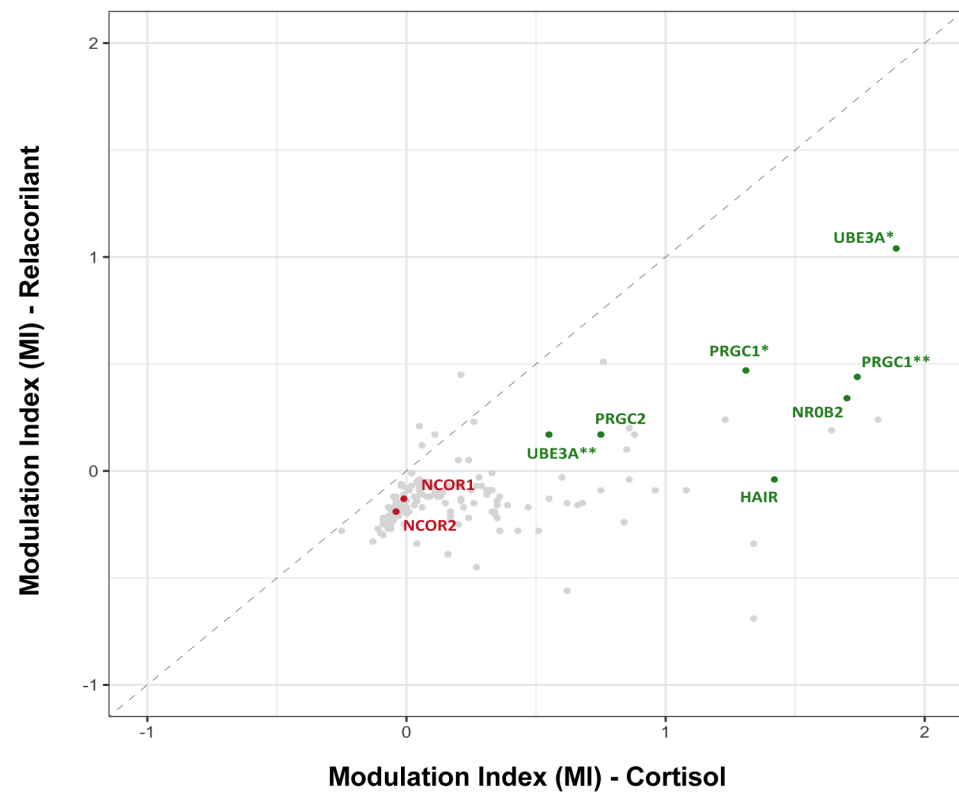

Supplement: Supplementary Figure 3. (A) DEX dose-response curve for Fkbp5, Gilz, Pomc and Nr3c1 in mouse AtT20 pituitary cells. All data are expressed as mean ±sem. The EC50 values were determined using GraphPad Prism 8 non-linear fitting. (B) Fold change-fold change plot of the interaction score of GR with nuc [file supplementary_figure_3.pdf]
