# Supplementary material for: Peripheral glucocorticoid receptor antagonism by relacorilant with modest HPA axis disinhibition
Source: J Endocrinol. 2022 Dec 22;256(2):e220263. doi: 10.1530/JOE-22-0263 (PMC9874980; doi:10.1530/JOE-22-0263)
Supplement: Supplementary table 1. Detailed sequences of all qPCR primers. [file supplementary_table_1.pdf]

**Gene**	**Assay**	**Order_number**	
Hprt	AtT20 cells	Mm03024075_m1	
GusB	AtT20 cells	Mm01197698_m1	
Gapdh	AtT20 cells	Mm99999915_g1	
Pomc	AtT20 cells	Mm00435874_m1	
Gilz	AtT20 cells	Mm01306210_g1	
Fkbp5	AtT20 cells	Mm00487401_m1	
Nr3c1	AtT20 cells	Mm00433832_m1	
**Gene**	**Assay**	**Forward primer (3'-5')**	**Reverse primer (3'-5')**
Fkbp5	Mouse	GCCGACTGTGTGTGTAATGC	CACAATACGCACTTGGGAGA
Gilz	Mouse	TGGCCCTAGACAACAAGATTGAGC	CCACCTCCTCTCTCACAGCAT
Mt2a	Mouse	CGCCTGCAAATGCAAACAATG	TCGGAAGCCTCTTTGCAGAT
Nr3c1	Mouse	CCCTCCCATCTAACCATCCT	ACATAAGCGCCACCTTTCTG
Pomc	Mouse	CGAGGCCTTTCCCCTAGAGT	CCAGGACTTGCTCCAAGCC
Mttp	Mouse	CTCTTGGCAGTGCTTTTTCTCT	GAGCTTGTATAGCCGCTCATT
Ucp1	Mouse	TCAGGATTGGCCTCTACGAC	TGCATTCTGACCTTCACGAC
Atgl	Mouse	ACAGTGTCCCCATTCTCAGG	TTGGTTCAGTAGGCCATTCC
Cyp11b1	Mouse	GTCAGATTCACTTGGGGGCA	GACATGCCCTCCCTGTATGG
Sgk1	Mouse	AGAGGCTGGGTGCCAAGGAT	CACTGGGCCCGCTCACATTT
18S	Mouse	AGGACCTGGAGAGGCTGAAG	CAGTGGTCTTGGTGTGCTGA
36B4	Mouse	GGACCCGAGAAGACCTCCTT	GCACATCACTCAGAATTTCAATGG
B2m	Mouse	TGACCGGCTTGTATGCTATC	CAGTGTGAGCCAGGATATAG
B-Actin	Mouse	AACCGTGAAAAGATGACCCAGAT	CACAGCCTGGATGGCTACGTA
